# Editorial: Regulation of fertility in livestock species

**DOI:** 10.3389/fgene.2024.1404237

**Published:** 2024-04-03

**Authors:** Hamayun Khan, Abdolrahman Khezri

**Affiliations:** ^1^ College of Veterinary Science, Faculty of Animal Husbandry and Veterinary Science, The University of Agriculture Peshawar Pakistan, Peshawar, Pakistan; ^2^ Faculty of Applied Ecology, Agricultural Sciences and Biotechnology, Inland Norway University of Applied Sciences, Hamar, Norway

**Keywords:** fertility, livestock species, gene expression and regulation, cRNA-seq analysis, differential gene expression, DNA methylation

Fertility is a complex trait influenced by several factors. Recently many different reproductive biotechnology techniques have been developed to achieve reproductive efficiency and consistency in livestock industries. Among those techniques, genomic selection, artificial insemination (AI), *in vitro* fertilization, semen sorting, and embryo transfer gained lots of attention among researchers. However, how those modern technologies affect the health and physiology of newly generated animals, is not well understood. Major advancements in -omics technologies (metabolomics, proteomics, transcriptomics, and genomics) have enabled high-throughput screening of a wide range of molecular and cellular dynamics in fertility molecules. These approaches also provide a means of detecting minute amounts of changes in molecules due to their higher sensitivity. Such attributes of these advanced methods are vitally important for innovative studies to produce new knowledge with transformational and translational values. Genome-wide association studies (GWAS) have been effective in applying dense genetic markers, such as single-nucleotide polymorphism (SNP) markers, to determine genomic regions associated with economically important phenotypes such as fertility. There is a need for new knowledge on the expression levels and functions of sperm RNA, proteins, and metabolites. The new knowledge can shed light on additional fertility markers that can be used in combination with scrotal circumference to predict the fertility of breeding bulls. As an economically important trait, fertility has become more important as there is an urgent need for more efficient, sustainable, and profitable production of food animals to feed the ever-increasing human population in the world.

Consequently, the current Research Topic was aimed and scientists in the relevant field across the world were invited to research the genetic and environmental regulation of fertility traits in livestock species in both male and female animals, as well as the genetic factors evaluated through molecular techniques including transcriptomics, proteomics, and metabolomics; the environmental factors evaluated through dietary, hormonal, or extenders, etc. used for the modulation of both male and female fertility (Khan et al., 2017; Haq et al., 2021). All-important molecular mechanisms such as transcriptional and post-transcriptional regulation of adipogenic marker genes. RNAs, transcription factors, candidate genes, SNPs, sequencing, DNA methylation, and DNA-protein interaction for the improvement of fertility traits were considered in this Research Topic. The Research Topic was accepted on 21 February 2023 and published on 2 March 2023 with the titled “Regulation of fertility in livestock species,” and closed on February 2024. A total of five manuscripts were published by 54 authors around the globe. Keeping in view the importance of regulation of fertility traits obtained from different livestock species, we received manuscripts regarding goats, Atlantic salmon (*Salmo salar* L.) dairy cows, and sheep. We can summarize the published articles with the following description against each group of manuscripts based on the target species of livestock.

The first paper submitted in this Research Topic was associated with the fertility of goats. In this manuscript, the researchers have explored comparative transcriptome profiling of granulosa cells (GCs) of high- and low-fertility goats, using scRNA-seq. These findings provided insights into the genetic basis of fertility in female goats and are an impetus to elucidate molecular ceRNA regulatory networks and functions of DEGs underlying ovarian follicular development. This study used a novel approach to combining various types of RNA as an integrated network in goat fertility. Analyses of scRNA-seq data resulted in the identification of 150 DEGs in goats with high versus low fertility. Among them, 80 genes were upregulated and 70 were downregulated. Moreover, 81 mRNAs/genes, 58 circRNAs, 8 lincRNAs, 19 lncRNAs, and 55 miRNAs, all well-known types of regulatory RNAs, were obtained from literature mining. Using circRNA–lincRNA–lncRNA–miRNA–mRNA ceRNA, a regulatory network was constructed and these identified RNAs were mainly associated with transcriptional regulatory activities and signaling receptor-binding activities in terms of MFs, as well as reproductive functions such as ovulation cycle, ovarian follicle development, growth, and differentiation cells based on BPs. Furthermore, our results are a valuable resource to elucidate molecular networks and the functions of DEGs underlying ovarian follicular development, and they increase the understanding of the genetic basis of high-versus low-fertility goats.

In this Research Topic, two papers were submitted regarding RNAs, transcription factors, candidate genes, SNPs, Sequencing, and DNA methylation. In one of the manuscripts, genomic DNA from Tan, Hu sheep and their F2 generation was bisulfite converted and subject to whole genome sequencing. Distinct genome-wide DNA methylation patterns were observed between Tan sheep and Hu sheep. Moreover, DNA methylated regions were significantly increased in the skeletal muscle Compared with Hu sheep, the methylation levels of actin alpha 1 (ACTA1), myosin heavy chain 11 (MYH11), Wiskott-Aldrich syndrome protein (WAS), vav guanine nucleotide exchange factor 1 (VAV1), fibronectin 1 (FN1) and Rho-associated protein kinase 2 (ROCK2) genes were markedly distinct in the Tan sheep. Furthermore, Gene Ontology analysis indicated that these genes were involved in myotube differentiation, myotube cell development, smooth muscle cell differentiation, and striated muscle cell differentiation. The findings from this study demonstrated that the ACTA1, MYH11, WAS, VAV1, FN1, and ROCK2 genes may exert regulatory effects on muscle development. Hence, the DNA methylation profiles provided new clues for deciphering epigenetic regulatory mechanisms involved in mutton development and identified novel candidate genes. In the second paper, the objective was to examine sperm quality parameters, fertilizing potential, metabolites, and DNA methylation in cold-stored and cryopreserved milt from Atlantic salmon (*S. salar* L.). This study aimed to investigate sperm quality parameters and fertilization potential of Atlantic salmon milt, stored cold and subsequently cryopreserved, using different storage conditions. Additionally, it was also the objective of the study to assess if analysis of milt metabolites and sperm DNA methylation signatures could be applicable to elucidate sperm quality and fertilization further following preservation to create contrasts for the exploration of the potential value of metabolomics and epigenetics on Atlantic salmon sperm and milt. The finding obtained from the study clarified successfully that storage duration and corresponding cryopreservation influenced metabolites and sperm DNA methylation signatures. Further studies should include shorter preservation intervals to reveal the possibility of applying an extended period of storage before cryopreservation.

The third group of manuscripts submitted was related to Endometrial DNA methylation signatures during the time of breeding in relation to the pregnancy outcome in postpartum dairy cows fed a control diet or supplemented with rumen-protected methionine. Post-calving metabolic stress is associated with a reduction of the fertility of high-producing dairy cows possibly by altering the expression of genes in the maternal environment via epigenetic modifications. Therefore, this study was conducted to identify endometrial DNA methylation marks that can be associated with pregnancy outcomes in postpartum cows at the time of breeding. In this study, we have characterized and identified endometrial DNA methylation landscapes in relation to the pregnancy outcomes in postpartum cows supplemented with RPM or fed a control diet. The results from the present study highlighted that either in the presence or absence of RPM supplementation, the postpartum cows that can be pregnant can be distinguished from those that resulted in no pregnancy based on their endometrial epigenome signatures at the time of breeding. These differences may indicate the presence of genome remodeling in the endometrial environment in expectation of the growing embryo. Furthermore, RPM supplementation has shown incremental effects on the DNA methylation patterns of cows that resulted in no pregnancy than those that became pregnant although supplementation of methionine altered the endometrial epigenome profile of both cow groups. In addition, a set of 19 differentially methylated genes were identified in all experiments and these genes might be fundamentally correlated with cow fertility status as well as methionine supplementation. These genes might be attractive candidates for further studies related to nutrigenomics for improving dairy cows. Eventually, these results may provide an opportunity to further navigate and screen promising endometrial DNA methylome markers which may be strongly associated with postpartum cow fertility at the time of breeding.

One paper submitted to the Research Topic was associated with Comprehensive transcriptomic analysis revealing the regulatory dynamics and networks of the pituitary-testis axis in sheep across developmental stages. The study aimed to explore the gap in the regulatory mechanism of the Spermatogenesis which is a complex process intricately regulated by the hypothalamic-pituitary-testis (HPT) axis. However, research on the regulatory factors governing the HPT axis has not yet been clarified. This study addresses this gap by conducting a comprehensive analysis of transcriptomes from the pituitary and testis tissues across various developmental stages, encompassing embryonic day (E120), neonatal period (P0), pre-puberty (P90), and post-puberty day (P270). Utilizing edgeR and WGCNA, we identified stage-specific genes in both the pituitary and testis throughout the four developmental stages. Notably, 380, 242, 34, and 479 stage-specific genes were identified in the pituitary, while 886, 297, 201, and 3,678 genes were identified in the testis. Subsequent analyses unveiled associations between these stage-specific genes and crucial pathways such as the cAMP signaling pathway, GnRH secretion, and male gamete generation. Furthermore, leveraging single-cell data from the pituitary and testis, we identified some signaling pathways involving BMP, HGF, IGF, and TGF-β, highlighting mutual regulation between the pituitary and testis at different developmental stages. This study sheds light on the pivotal role of the pituitary-testis axis in the reproductive process of sheep across four distinct developmental stages. Additionally, it delves into the intricate regulatory networks governing reproduction, offering novel insights into the dynamics of the pituitary-testis axis within the reproductive system.
